# Green synthesis of nisin-loaded pectin based silver nanoparticles and their antimicrobial and biosafety assessment

**DOI:** 10.1186/s11671-026-04498-6

**Published:** 2026-04-09

**Authors:** Yu-qiu Jiang, Run-sheng Xie, Pei-jun Li, Rui-min Zhong, Jian-hua Zhu, Hui Tang, Xin-jie Li

**Affiliations:** 1 College of Chemistry and Bioengineering, Guilin University of Technology, Guilin, 541004 China; 2 Guangdong Provincial Key Laboratory of Utilization and Conservation of Food and Medicinal Resources in Northern Region, College of Food Science & Technology, Shaoguan University, Shaoguan, 512005 China

**Keywords:** Silver nanoparticles, Nisin, Conjugate, Antibacterial, Biosafety

## Abstract

Antibacterial ability and biosafety of silver nanoparticles (AgNPs) is attracting more and more attention. In this work, nisin was loaded on the surface of pectin-based AgNPs (P-AgNPs), and NP-AgNPs was synthesized by using a one-pot method. The nanomaterials were characterized by using the following methods, TEM showed that the average diameters were changed from 21.79 to 25.35 nm, FT-IR indicated that Ag^+^ was reduced by electrostatic interaction, XPS showed that nisin was conjugated on the surface of AgNPs, and Zeta potential assays showed the changes of surface potential. It was worth noting that NP-AgNPs had excellent antibacterial capacity, the minimum inhibitory concentration (MICs) reached 16.7 and 33.3 µg/mL against *E. coli* and *S. aureus*, respectively, which were 32 and 16 times lower than those of P-AgNPs. The experimental results indicated that the loading of nisin increased the zeta potential of NP-AgNPs, which made it easier to bind to the bacterial cells. Antibacterial mechanism indicated that NP-AgNPs caused cell membrane and cell wall damage, and produced more ROS, which resulted in bacterial lysis and leakage. Furthermore, NP-AgNPs showed higher hemocompatibility, lower cytotoxicity, and higher inhibitory effects on tumour cells. Overall, NP-AgNPs have the potential to be used in pharmaceutical and food industry.

## Introduction

Antibiotic resistance is recognized as one of the most pressing health threats by the World Health Organisation (WHO), underscoring the urgent necessity for the development of new and effective alternative antimicrobial agents [1]. The physicochemical properties of metallic nanomaterials (MNPs) can be manipulated to produce effective antimicrobial agents, offering a novel approach to combat antibiotic resistance [2]. Silver nanoparticles (AgNPs) have garnered prominence due to their broad-spectrum antimicrobial properties and multifaceted mechanisms of action [3], making them highly favored in various sectors such as the food and pharmaceutical industries [4, 5]. Nevertheless, traditional methods for synthesis of AgNPs frequently involved toxic chemical reagents and necessitate complex equipment, which posed significant risks to both environmental safety and human health [6].

To address these limitations, plant extracts and biomacromolecules have been chosen for the green synthesis of AgNPs [7, 8]. Pectin, a natural polyanionic polysaccharide derived from fruits, comprised of α-1,4 glycosidic linkages connecting units of galacturonic acid, and proved to be an excellent biomaterial for the synthesis of MNPs [9]. In our previous research, pectin-based AgNPs (P-AgNPs) were prepared by silver nitrate and alkali hydrolysis of pectin under microwave assistance, and the products had broad-spectrum antimicrobial properties [10]. However, the antibacterial efficiency of AgNPs still needed to be improved, and its cytotoxicity also needed further consideration.

Typically, the antimicrobial efficiency and biosafety of AgNPs were closely associated with their size, shape, and surface coating [11, 12]. For instance, AgNPs featured with smaller sizes and certain ligand layers could better interact with the bacteria, enhancing antimicrobial efficiency [11]. On the other hand, it was reported that smaller-sized AgNPs might increase the toxic effects on mammalian cells [12]. In light of this, researchers found that the conjugation of AgNPs with organic molecules enhanced both antibacterial efficacy and biosafety.

Antimicrobial peptides (AMPs) are natural components produced by the immune systems of organisms to protect themselves against pathogenic invasion. Usually, they exhibit broad-spectrum antibiotic activity against bacteria, viruses, and fungi [13]. Recent studies have demonstrated that certain AMPs exhibit synergistic effects when combined with conventional antibiotics. This combined approach could effectively kill drug-resistant pathogens, prevent the emergence of resistance, and significantly enhance the therapeutic efficacy of antibiotics [14]. However, the susceptibility of AMPs to degradation as natural antimicrobial agents limited their development and application. It has been reported that AMPs exhibited enhanced stability and improved antimicrobial efficiency when integrated with nanotechnology [15]. Previous studies have shown that AMPs can enhance the antimicrobial efficiency and biosafety of AgNPs [16]. Therefore, the combination of AMPs with AgNPs may be a win-win strategy to address the limitations inherent in both. Nisin, a small cationic peptide (3510 Da, comprising 34 amino acids) produced by *Lactococcus lactis subsp*, possesses antimicrobial, anti-inflammatory, and antitumor activities [17]. Furthermore, it is non-toxic to humans and is commonly used as a food additive to inhibit the proliferation of spoilage bacteria in food products [17, 18]. In recent years, nisin has been extensively employed in the field of biomedicine, showed good effects in oral care and antitumor aspects [19]. However, in order to achieve the desired bacteriostatic effect, higher concentrations of nisin was required. In addition, nisin did not exhibit inhibitory activity against Gram-negative bacteria [17].

To overcome the shortcomings of nisin and AgNPs, we synthesized a novel nisin loaded pectin-silver nanoparticles (NP-AgNPs) by using a one-pot method. To our knowledge, this is the first report on the bioconjugation of P-AgNPs with nisin. Firstly, several characterizations of NP-AgNPs were performed to elucidate the conjugation mechanism between nisin and AgNPs. Secondly, the antimicrobial properties of NP-AgNPs were tested and the antimicrobial mechanism was revealed. Finally, the biosafety and antitumour activity of NP-AgNPs were evaluated by haemolysis and cytotoxicity assays.

## Materials and methods

### Materials

Citrus peel pectin (P9135, containing 74.0% galacturonic acid), Nisin (≥ 1108 IU/mg), and Silver nitrate (AgNO_3_, ≥ 99.8%, AR) were purchased from Tokyo Chemical Industry, Shanghai Bide Pharmaceutical Technology and Xilong Scientific Co., Ltd., respectively. Protein, ROS and Cell Counting Kit-8 (CCK-8) were acquired from Shanghai Biyuntian Biotechnology and Dongren Chemical Technology Co., Ltd. Every reagent utilized in the study was of analytical grade. *Escherichia coli* (strain 133264) and *Staphylococcus aureus* (strain 186335) were obtained from the Beijing Beina Chuanglian Biotechnology Research Institute in China. Human colon cancer cells HCT-116, mouse fibroblast cells NIH-3T3, and fresh mouse whole blood were all purchased from Wuhan Procell Biotechnology Co., Ltd.

### Synthesis of the AgNPs bioconjugates

Followed the previously described synthesis method [10], 4 mL of AgNO_3_ solution (0.5 mol/L) was vortex-mixed with 10 mL of nisin solution (at 0, 5, 10, and 12.5 mg/mL) for 10 min used a magnetic stirrer. Pectin powder (0.4 g) was dissolved in 200 mL of a 1 g/L NaOH aqueous solution at room temperature. The nisin-AgNO_3_ mixture was then introduced dropwise, and the reaction was conducted at 65 °C with stirring at 300 rpm for 20 min to produce an AgNPs bioconjugate solution. The reaction mixture underwent centrifugation for 20 min at 10,000 rpm, and the precipitate was washed twice to eliminate any unbound substance and finally dried for 8 h at 50 °C to yield the AgNPs powder. The AgNPs were designated as P-AgNPs, NP-AgNPs1, NP-AgNPs2, and NP-AgNPs3, corresponding to the varying nisin concentrations of 0, 5, 10, and 12.5 mg/mL, respectively.

### Detection and characterization of AgNPs

According to the previous method [20], the colloidal solution of AgNPs was analyzed with a UV-vis spectrophotometer, transmission electron microscopy (TEM), and Zeta Size Nano. Dried AgNPs powders underwent further examination used scanning electron microscopy (SEM), Fourier transform infrared spectroscopy (FT-IR), X-ray diffraction (XRD), and X-ray Photoelectron Spectroscopy (XPS). The thermal properties were evaluated used a thermogravimetric analyzer (TGA) under a nitrogen environment, ranged in temperature from 25 to 800 °C and a 10 °C/min heated rate.

### Antibacterial tests

#### Bacteria culture

Followed previously established protocols [10, 21], LB (Lennox broth) medium was sterilized for 20 min at 121 °C. After sterilization, both bacteria were inoculated into the medium and incubated with shaking for 18–24 h at 37 °C. The activated bacteria solutions were diluted with sterile water and counted by a hemocytometer to ensure a concentration of 10^7^ CFU/mL.

#### In vitro evaluation of antibacterial activity

The minimum inhibitory concentration (MIC) of the AgNPs bioconjugates against *E. coli* and *S. aureus* was determined used the micro broth dilution technique [20]. The AgNPs powder was suspended in LB broth (1 mL) at a 3.2 mg/mL concentration. A series of dilution gradients were then prepared with 0, 0.05, 0.1, 0.2, 0.4, 0.8, 1.6, and 3.2 mg/mL concentrations. 200 µL of LB broth, 50 µL AgNPs bioconjugates suspension, and 50 µL bacterial suspension were thoroughly mixed and placed into well plates. The resulted concentrations of AgNPs bioconjugate in the wells were 0, 8.3, 16.7, 33.3, 66.7, 133.3, 266.7, and 533.3 µg/mL. The absorbance of the cultures at 600 nm was measured every 3 h used a microplate reader.

### Antibacterial mechanism

#### Bacteria morphology observation

The AgNPs bioconjugate powder was mixed with bacteria suspensions and incubated at 37 °C with shaking. After 6 and 24 h of incubation, the cultures were centrifuged at 6000 rpm for 5 min to collect the bacterial cell pellets. After fixed in glutaraldehyde (2.5%, w/w) for 4 h at 4 °C, the cells were sequentially dehydrated for 15 min with 30, 50, 70, 90, and 100% ethanol concentrations. The samples were then lyophilized and examined by using SEM.

#### Intracellular protein leakage and ROS level analysis

The Bradford assay was employed to measure the leakage of intracellular proteins from bacteria subjected to AgNPs stress [22]. The BCA protein assay kit was used to determine the concentration of extracellular protein in the bacterial suspension. In the MIC experiment, bacteria were incubated at 37 °C for 24 h after being exposed to 533.3 µg/mL of AgNPs.

The variation in intracellular ROS level was determined used fluorescence spectrometer. The wavelengths of excitation and emission were adjusted to 488 and 525 nm, respectively. After being exposed to 533.3 µg/mL of AgNPs, bacteria underwent incubation for 0 and 6 h at 37 °C, and then centrifuged at 2400 rpm at 4 °C. After collection of the supernatant (2 mL), a diluted DCFH-DA reagent (3 mL, 1:1000 in LB broth) was added. The mixture was centrifuged under the same conditions after it had been incubated for 1 h at 37 °C. The fluorescence intensity of the supernatant was then measured after 0 and 6 h, respectively.

### Biosafety evaluation

#### Hemolysis assessment

Hemocompatibility testing was referred to previous report with slight modifications [23]. Fresh mouse whole blood (1 mL) was mixed thoroughly with 9 mL of PBS buffer (pH 7.4). Next, AgNPs suspension (0.5 mL) was introduced to 0.1 mL of erythrocytes in each centrifuge tube. The 0.5 mL of PBS buffer and 0.1% Triton X-100 solution were used as the positive and negative controls, respectively. After incubated 1 h at 37 °C, the samples were centrifuged at 1500 rpm for 10 min. Photographs were captured to analyze hemolysis in each group. Absorbance in the supernatant was analyzed at 540 nm using a microplate reader, and the rate of hemolysis was determined as follows [23]:

$$Hemolysis\left(\%\right)=\frac{({A}_{i}-{A}_{j})}{({A}_{0}-{A}_{j})}\times100\mathrm{\%}$$

where A_i,_ A_j_, and A_0_ represent the absorbance values of the sample, positive control, and control, respectively.

#### Cytotoxicity assessment

Cytotoxicity testing was referred to previous report with slight modifications [4], the cytotoxicity of nisin, P-AgNPs, and NP-AgNPs against NIH-3T3 and HCT-116 was determined using a CCK-8 kit. Briefly, cell suspension (100 µL) was placed into a well plate, and the cells were pre-cultured to a suitable concentration of 5 × 10^3^ cells/well. The cells were then co-cultured with samples solution (10 µL) at various doses (0.2, 0.4, 0.8, 1.6, and 3.2 mg/mL) in each well for 24 h. Subsequently, CCK-8 solution (10 µL) was introduced to each well, and the incubation was continued for an additional 2 h. Absorbance at 450 nm was determined used a microplate reader for each sample group. The group containing medium and cells as the control, and containing medium and CCK-8 were used as blank. The cell survival rate was calculated as follows [4, 1]:

$$Cellsurvival\left(\%\right)=\frac{({A}_{s}-{A}_{b})}{({A}_{c}-{A}_{b})}\times100\%$$

Where A_s_, A_b_, and A_c_ indicate the absorbance values of the sample, the blank group, and the control group, respectively.

### Statistical analysis

All data are expressed as mean ± SD. Data statistics and plotting were performed using Origin and GraphPad Prism 8.0.2 software. Statistical analysis was performed by one-way ANOVA, and *p* < 0.05 was considered statistically significant (lowercase letters between different treatments indicate *p* < 0.05; **p < 0.005, ****p < 0.0001).

## Results and discussion

### Synthesis and characterization of AgNPs bioconjugates

**Fig. 1:**
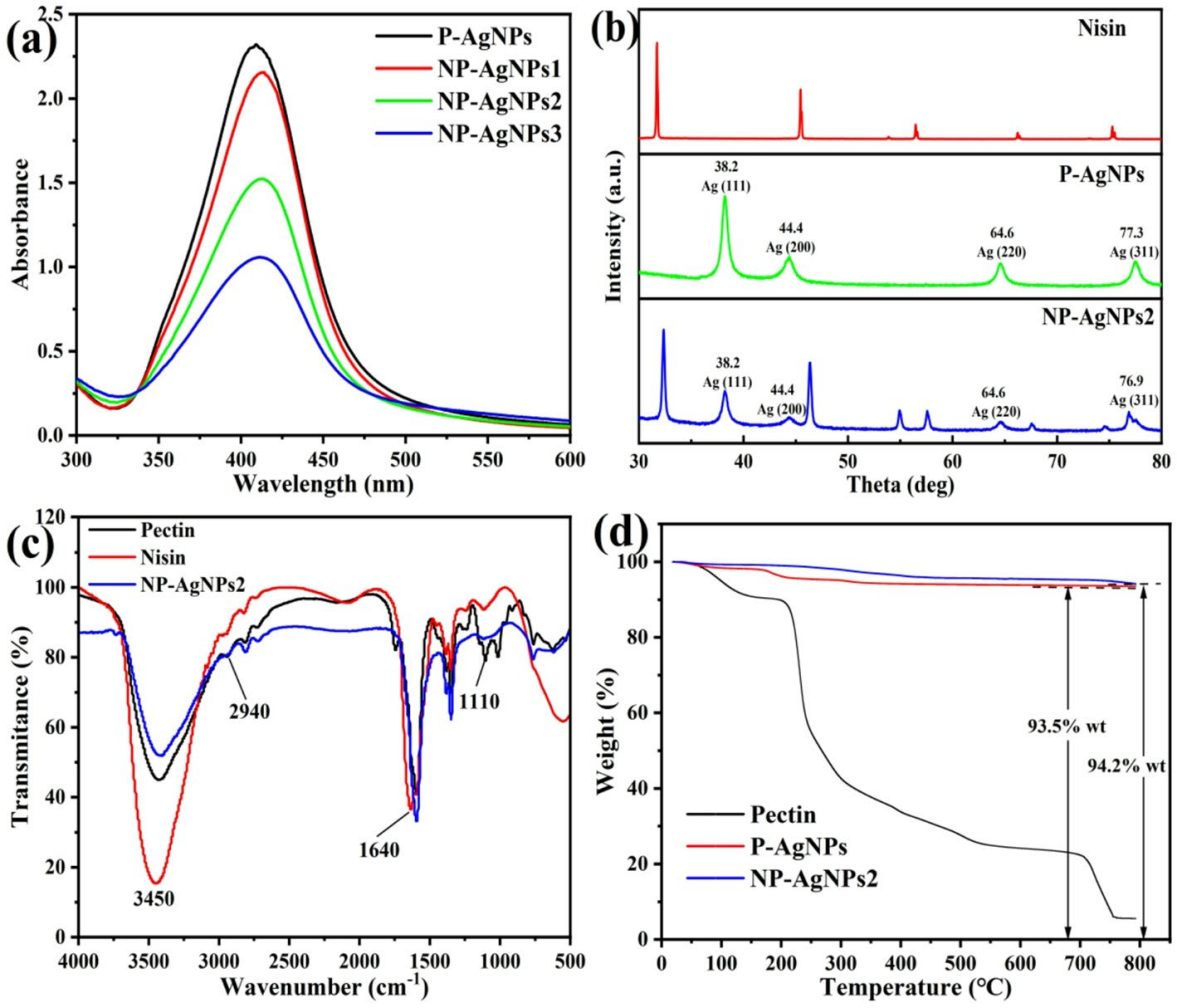
UV-vis absorption spectra of P-AgNPs, NP-AgNPs1, NP-AgNPs2 and NP-AgNPs3 (**a**); XRD analysis of nisin, P-AgNPs and NP-AgNPs2 (**b**); FT-IR specta of NP-AgNPs2, pectin and nisin (**c**); TGA of pectin, P-AgNPs and NP-AgNPs2 (**d**)

UV-visible absorption spectroscopy is commonly utilized for the characterisation of metal nanoparticles, with AgNPs typically exhibiting absorption peaks in the range of 400–450 nm [24]. Figure 1a showed that absorption peaks of the samples were detected around 410 nm, confirming the presence of AgNPs in the solution. Notably, NP-AgNPs exhibited a shift in UV-vis absorption towards longer wavelengths when compared to P-AgNPs, which might be attributed to the larger size of the nanoparticles. These findings are consistent with the results reported by Golubeva et al. [25], who investigated the bioconjugation of AgNPs with the peptide G Bac 34. Furthermore, the intensity of the absorption peak decreased gradually, which was due to an increase in the mass fraction of nisin in the solution.

The crystalline characteristics of the nanoparticles were assessed through XRD analysis (Fig. 1b). The XRD patterns of P-AgNPs and NP-AgNPs2 exhibited characteristic peaks at approximately 38.2°, 44.4°, 64.6°, and 76.9°, respectively, which correspond to the lattice planes [111], [200], [220], and [311] of metallic silver, respectively. This results are consistent with previous reports and indicate that the AgNPs possess a face-centered cubic crystal structure [26]. In addition, NP-AgNPs2 showed multiple non-Ag diffraction peaks in the range of 30–80°. The XRD detection of nisin showed that the characteristic peaks in the range of 30–80° had extremely high similarity with the non-Ag diffraction peaks of NP-AgNPs2, indicating that the non-Ag diffraction peaks are caused by the crystallization characteristics of the nisin. It is worth noting that the non-Ag diffraction peaks of NP-AgNPs2 shifted and changed in intensity compared with nisin, indicating that there was an electrostatic interaction between nisin and AgNPs.

The functional groups of pectin, nisin, and NP-AgNPs were analyzed by using FT-IR. As illustrated in Fig. 1c, significant intensity changes of the -OH (3450 cm^− 1^), -CH_3_ (2940 cm^− 1^), C = O (1640 cm^− 1^) and C–O–C (1110 cm^− 1^) groups were observed. The FT-IR of NP-AgNPs2 showed that the stretching vibration of the -OH group was weakened, which was attributed to the reaction with Ag^+^; the asymmetric C–H stretching of the -CH_3_ group disappeared, indicating that Ag^+^ replaced the -CH_3_ group during the synthesis process [27]; the decrease in the peak intensities of the C=O and C–O–C groups suggested that these two functional groups participated in the reduction reaction of Ag^+^. In summary, the FT-IR results indicated that pectin and nisin were involved in the synthesis of NP-AgNPs.

According to TGA analysis, pectin exhibited three distinct stages of weight loss from 50 to 800 °C (Fig. 1d). The initial temperature range of 50–170 °C was associated with the evaporation of water. The subsequent range of 170–560 °C corresponded to the thermal decomposition of pectin, and leaded to significant mass loss and the formation of byproducts such as solid charcoal. From 560 to 800 °C, pyrolysis of the charcoal leaded to a more gradual mass loss [28]. In contrast, P-AgNPs demonstrated a noticeably slower rate of mass loss during 170–560 °C phase, which could be attributed to the thermal decomposition of pectin present on their surface. Figure 1d showed that combustion residues of P-AgNPs and NP-AgNPs2 were 93.5 and 94.2% (w/w), respectively, indicating that the conjugation of nisin improved the AgNPs’ thermal stability.

**Fig. 2:**
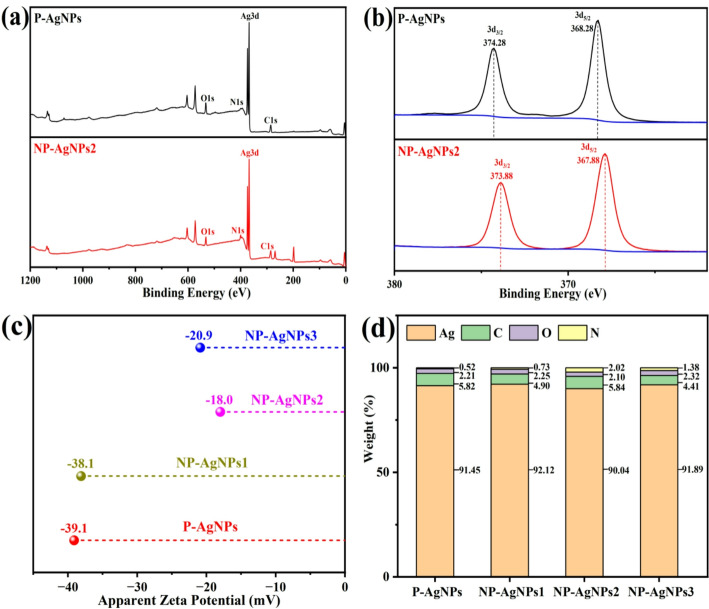
XPS full spectrum (**a**) and Ag spectrum (**b**) of P-AgNPs and NP-AgNPs2; Zeta potential (**c**) and EDS analysis (**d**) of P-AgNPs, NP-AgNPs1, NP-AgNPs2 and NP-AgNPs3

The interaction between nisin and AgNPs was investigated using XPS. As shown in Fig. 2a, characteristic peaks for Ag, C, O, and N elements were observed in the full spectra of P-AgNPs and NP-AgNPs2. Upon magnification of the Ag3d peak (Fig. 2b), the binding energies of Ag 3d_3/2_ and Ag 3d_5/2_ for P-AgNPs were found to be 374.28 eV and 368.28 eV, respectively; while for NP-AgNPs2, they were 373.88 eV and 367.88 eV, respectively. Compared to P-AgNPs, the binding energies of Ag3d in NP-AgNPs2 decreased, which could be attributed to the incorporation of nisin, resulting in a positive charge on the AgNPs [17]. This increase in outer electron density enhanced the shielding effect on the inner electrons, causing the binding energy to shift towards lower energy levels [7]. Figure 2c shows that the Zeta potentials of P-AgNPs, NP-AgNPs1, NP-AgNPs2, and NP-AgNPs3 were − 39.1, − 38.1, − 18.0, and − 20.9 mV, respectively. The changes in potential could be attributed to electrostatic interactions, indicating that the surface negative charge of the AgNPs was neutralised, leading to an increase in potential [29]. Both XPS and Zeta potential analyses demonstrated that nisin formed a stable conjugate with AgNPs through electrostatic interactions. Notably, NP-AgNPs2 exhibited the highest zeta potential among the test samples, which might be attributed to its strongest binding interaction with nisin. EDS data indicated that the Ag content in the four samples exceeded 90%, confirming that Ag was the predominant component (Fig. 2d). In addition, NP-AgNPs2 showed the highest nitrogen (N) content (2.02%, w/w), which indicated that more nisin was conjugated on the surface of NP-AgNPs2 [17].

**Fig. 3:**
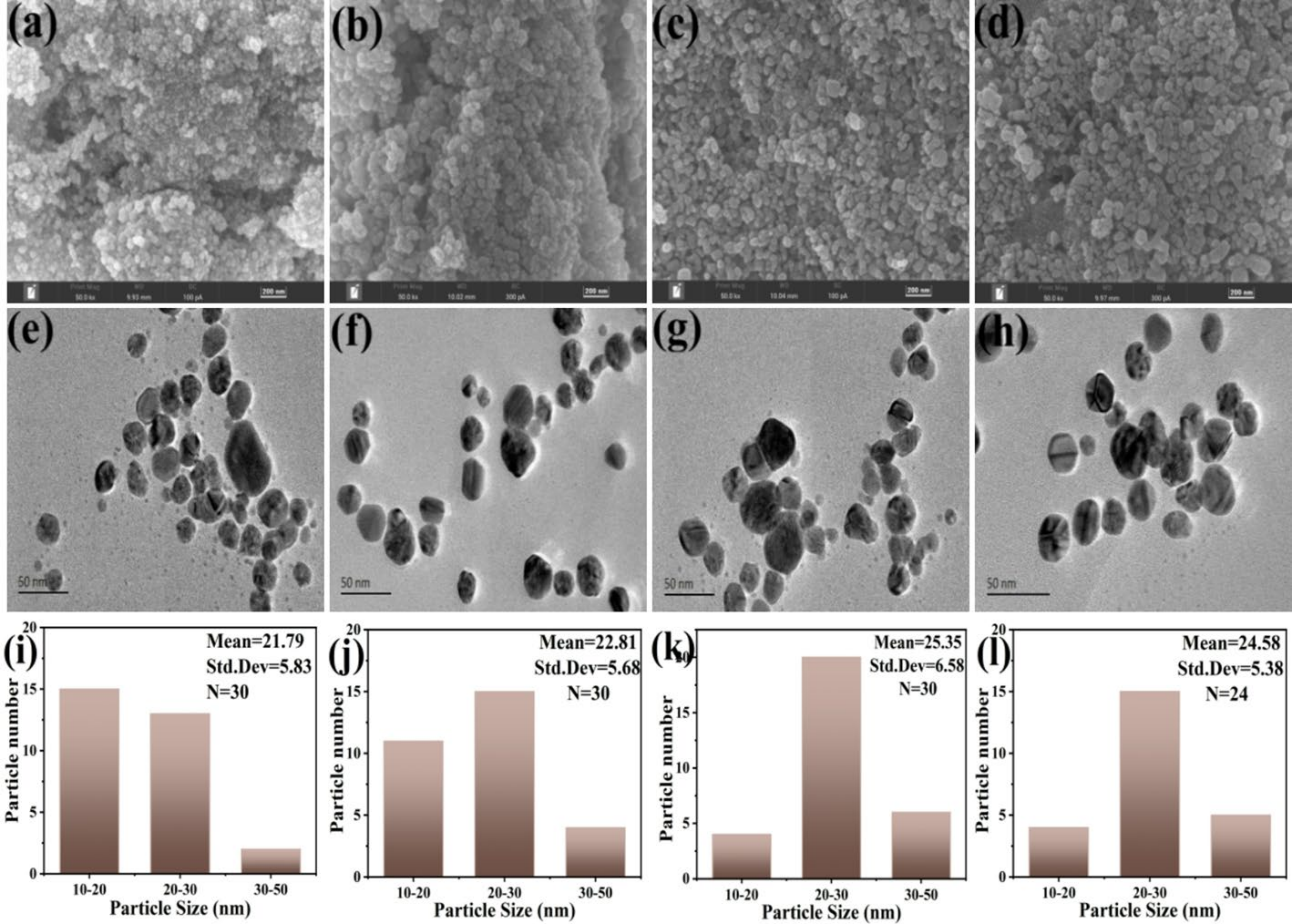
SEM images (**a**,** b**,** c**,** d**), TEM images (**e**,** f**,** g**,** h**), and size distribution (**i**,** j**,** k**,** l**) of P-AgNPs, NP-AgNPs1, NP-AgNPs2 and NP-AgNPs3, respectively

The distribution, morphology, and size of the nanoparticles were examined by using SEM and TEM analysis. As shown in Fig. 3a-h, SEM and TEM images revealed that the AgNPs were evenly distributed without significant aggregation, with all samples exhibiting a regular ellipsoidal shape. Size distribution indicated that the average diameters of P-AgNPs, NP-AgNPs1, NP-AgNPs2, and NP-AgNPs3 were 21.79 ± 3.42, 22.81 ± 2.73, 25.35 ± 2.46, and 24.58 ± 3.88 nm, respectively (Fig. 3i-l). These results meant that the conjugation of AgNPs with nisin resulted in an increase in the particle size, which was consistent with previous reports [15].

### Antimicrobial activity

**Fig. 4:**
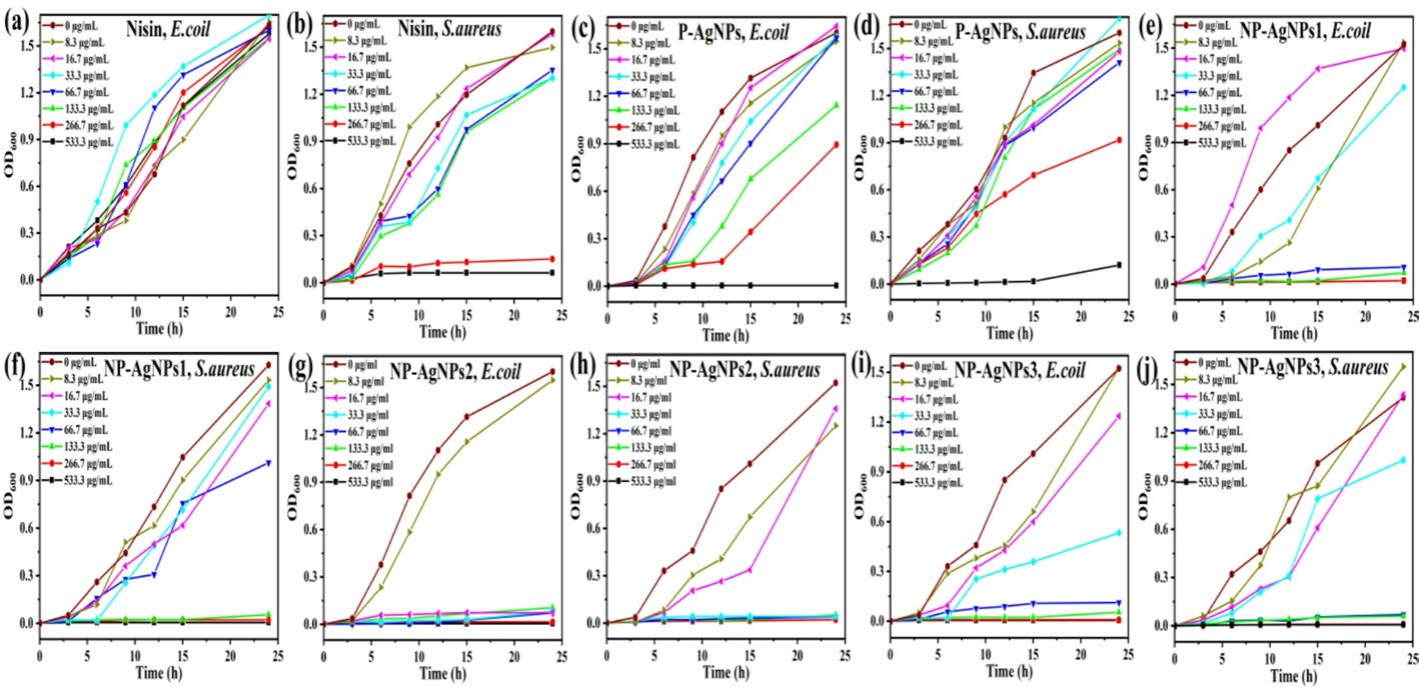
The growth curves of *E. coli* and *S. aureus* under different concentrations of nisin (**a**,** b**), P-AgNPs (**c**,** d**), NP-AgNPs1 (**e**,** f**), NP-AgNPs2 (**g**,** h**) and NP-AgNPs3 (**i**,** j**) after 24 h, respectively

The growth kinetics of *E. coli* and *S. aureus* under different concentrations of antimicrobial materials were evaluated using the micro broth dilution technique [20]. As shown in Fig. 4a and b, at the concentration gradient from 0 to 533.3 µg/mL, nisin exhibited no inhibitory effect on *E. coli*, while the MIC for *S. aureus* was 266.7 µg/mL. The MIC values of P-AgNPs against both bacterial strains was 533.3 µg/mL (Fig. 4c and d). Interestingly, NP-AgNPs demonstrated significantly enhanced antibacterial activity at concentrations of 16.7–66.7 µg/mL and 33.3–133.3 µg/mL, respectively (Fig. 4e-g). In particular, NP-AgNPs2 exhibited the best antibacterial activity, with MIC values of 16.7 and 33.3 µg/mL against *E. coli* and *S. aureus*, respectively, representing an increase of 32 and 16 times compared to P-AgNPs.

The results indicated that the combination of nisin and P-AgNPs improved their respective antimicrobial capabilities and antimicrobial spectrum, and the conjugate NP-AgNPs had a strong killing effect on both Gram-positive and negative bacteria. This phenomenon could be explained that the nisin modified the surface properties of AgNPs, and produced synergistic antibacterial effects against both *E. coli* and *S. aureus*.

### Antimicrobial mechanism

**Fig. 5:**
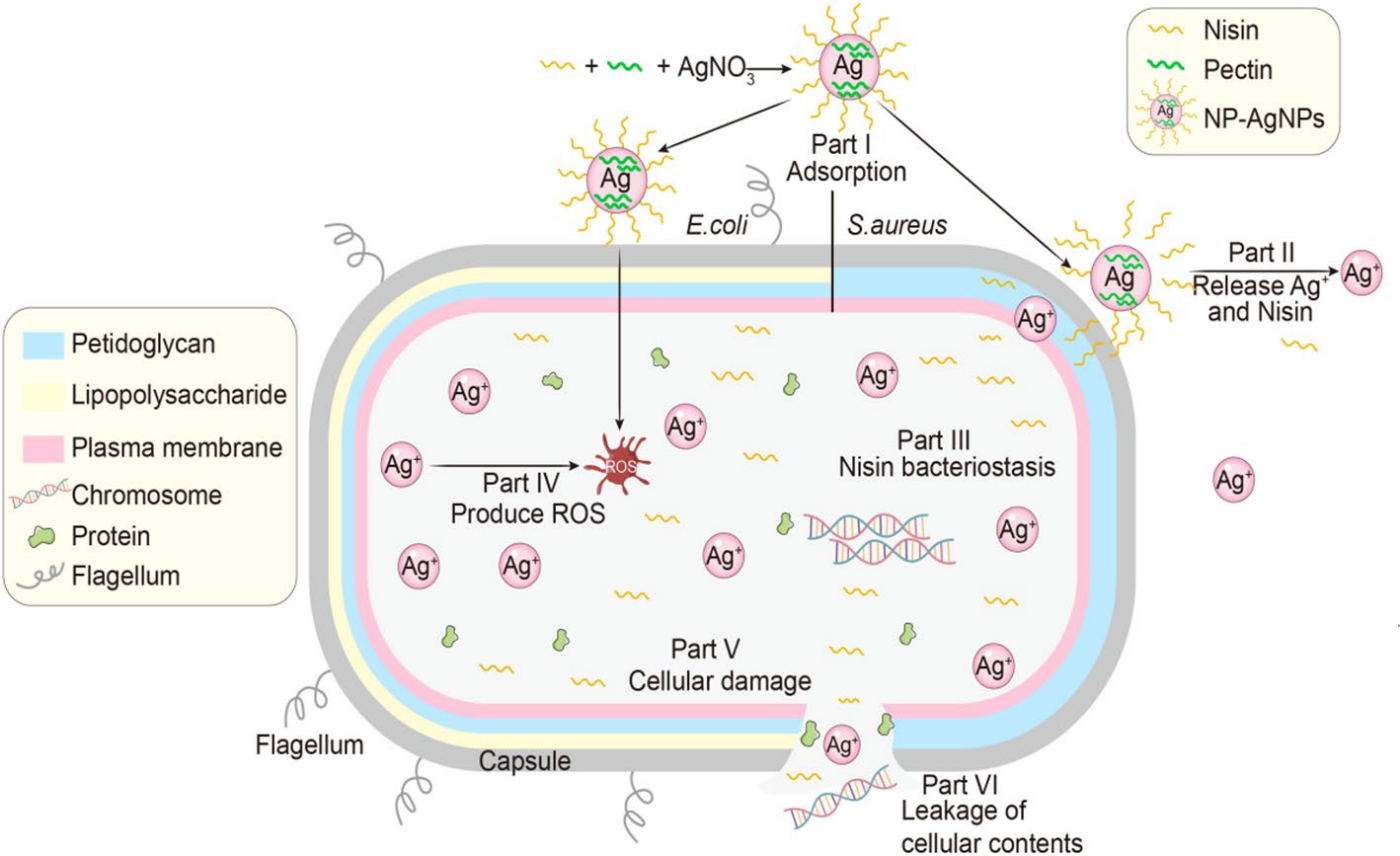
Schematic representation of the interaction between NP-AgNPs and bacterial cells

Figure 5 illustrates the interaction pathways between NP-AgNPs and bacterial cells. Initially, NP-AgNPs tightly adsorb onto the surface of bacterial cells (Part I). Subsequently, free Ag^+^ and nisin are released, interacting with the bacterial cell wall and cell membrane, thereby entering into the bacterial cells (Part II). Furthermore, nisin inhibits the activity of proteases, and disrupts the normal metabolism of bacteria; in addition, Ag^+^ generates reactive oxygen species (ROS), which causes bacterial cytotoxicity. Concurrently, both of nisin and AgNPs can interfere with the synthesis of DNA and RNA, halting bacterial growth and replication (Parts III and IV) [30]. Ultimately, under the combined action of multiple mechanisms, the bacterial cell wall is compromised, the integrity of the cell membrane is disrupted (part V), and the contents of the bacteria are leaked out from bacterial cells (Part VI)

#### Effects of AgNPs bioconjugates on bacteria morphology

**Fig. 6:**
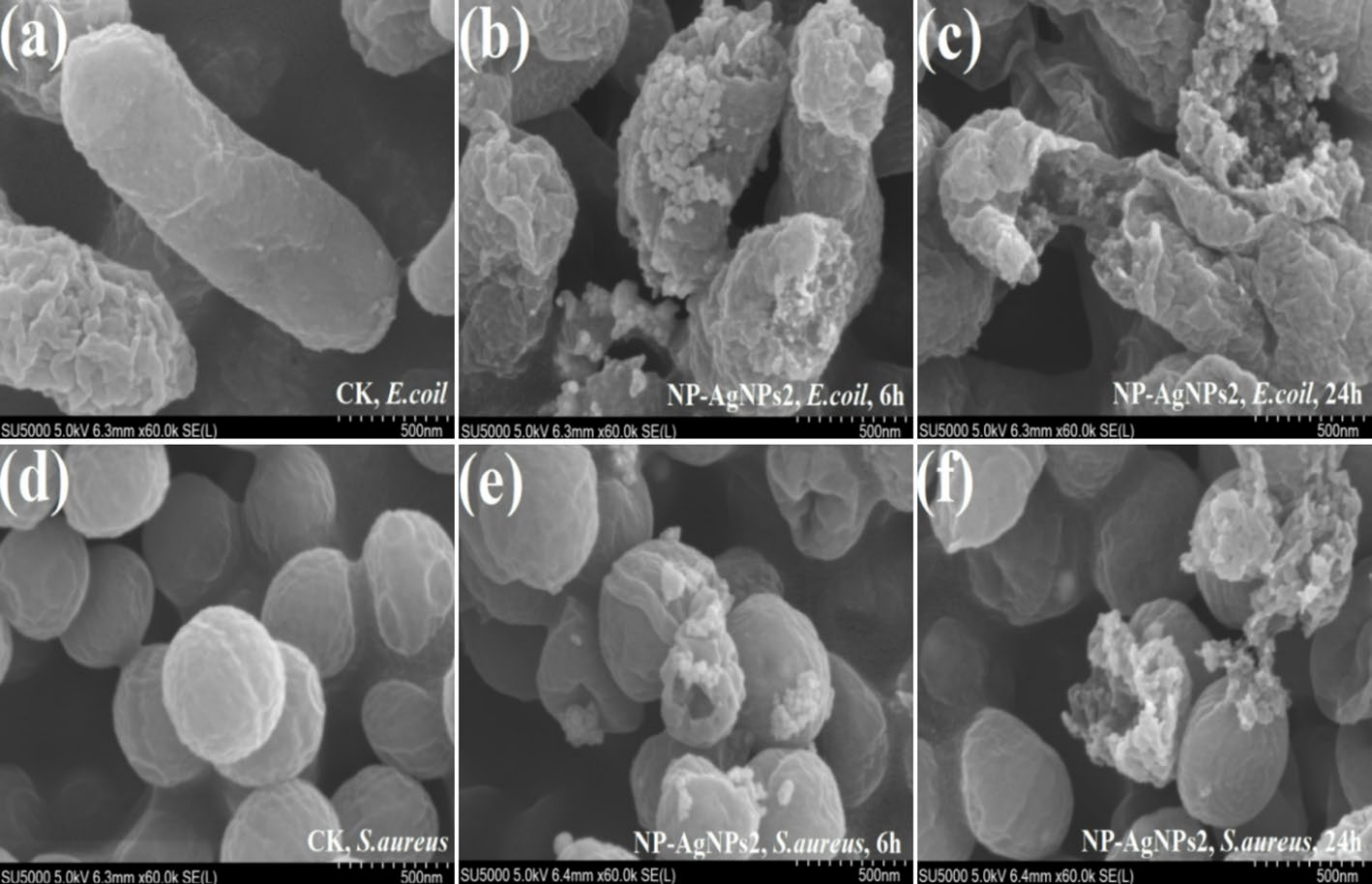
SEM images for *E. coli* (**a**,** b**,** c**) and *S. aureus* (**d**,** e**,** f**) under NP-AgNPs2 treatment for 6 and 24 h

As illustrated in Fig. 6, SEM images revealed the morphological changes in *E. coli* and *S. aureus* before and after NP-AgNPs2 treatment. Untreated *E. coli* retained its typical rod shape, while *S. aureus* maintained its natural spherical form (Fig. 6a and d). After 6 h of co-cultivation, NP-AgNPs2 adhered to the surface of cells and disrupted their structure, causing the cell walls to shrink and collapse (Fig. 6b and e). After 24 h of co-culture, bacteria suffered more severe damage, and most cells died (Fig. 6c and f). This indicated that NP-AgNPs disrupted the bacterial cell membranes and cell walls seriously.

#### Effects of AgNPs bioconjugates on bacterial cells

**Fig. 7:**
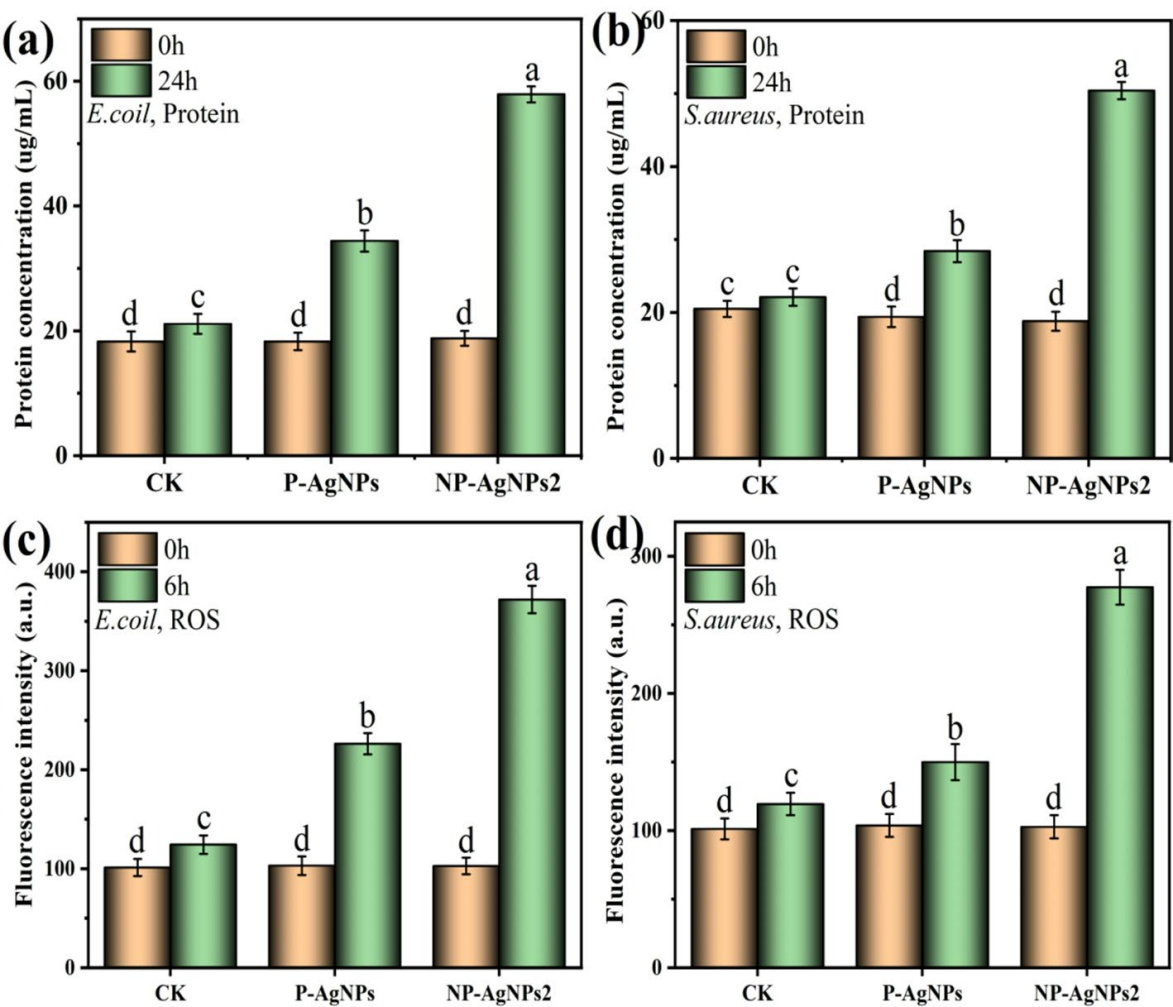
Protein leakage (**a**,** b**) and ROS content (**c**,** d**) in *E. coli* and *S. aureus* cells followed P-AgNPs and NP-AgNPs2 treatment

To further validate the bacterial cell damage under AgNPs stress, the leakage of intracellular contents from *E. coli* and *S. aureus* was assessed. As shown in Fig. 7a and b, only a minimal amount of protein leakage was observed in the blank group after 24 h of incubation. However, after AgNPs treatment for 24 h, the bacterial protein leakage increased significantly compared to the blank group. Taking *E. coli* as an example, after 24 h of treatment with P-AgNPs and NP-AgNPs2, the concentrations of extracellular proteins increased by 87.9 and 207.9%, respectively. As for *S.aureus*, the concentrations of extracellular proteins increased by 46.8 and 168.1%, respectively.

The antibacterial activity of AgNPs is commonly attributed to their ability to generate ROS [31]. As shown in Fig. 7c and d, the ROS produced under the stress of P-AgNPs and NP-AgNPs2 increased 119.3 and 269.2% for *E. coli*, and 45.4 and 170.1% for *S. aureus*, respectively. The former produces more ROS than the latter, which may also be explained why NP-AgNPs have stronger antimicrobial ability. It is generally believed that the AgNPs adhered to the cell membrane and affected membrane permeability, subsequently, penetrated the cell to generate reactive oxygen radicals [32]. The above results indicated that NP-AgNPs showed a stronger destructive ability toward bacteria than P-AgNPs.

### Biosafety

**Fig. 8:**
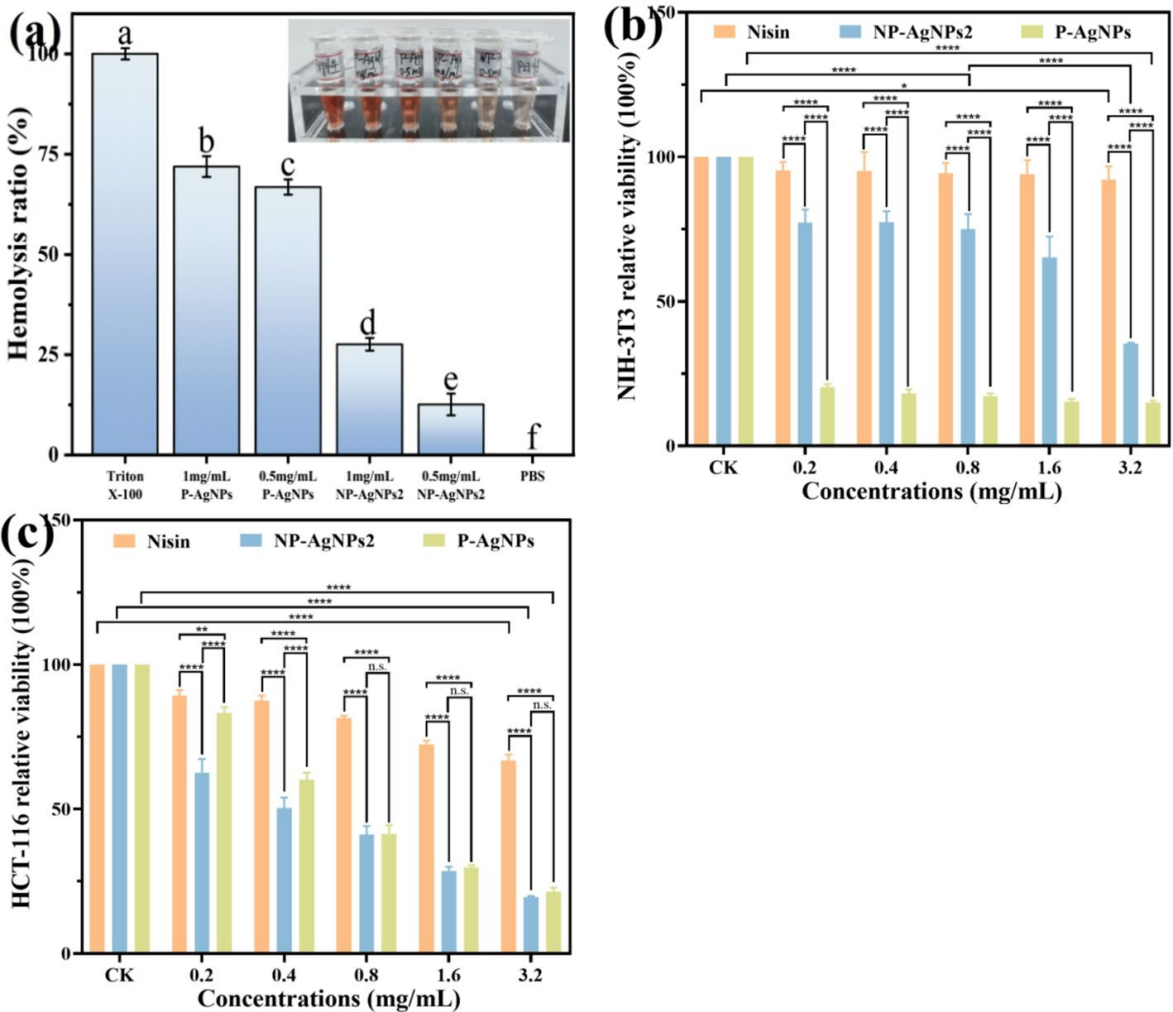
Effects of P-AgNPs and NP-AgNPs2 samples on the hemolysis ratio of fresh mouse red blood (**a**), and the activities of mouse fibroblast cells NIH-3T3 (**b**) and cancer cells HCT-116 (**c**), respectively

It has been reported that AgNPs exhibited toxicity to mammalian cells when the concentration exceeded a certain threshold, necessitating a balance between their therapeutic potential and adverse effects [33]. In this work, we evaluated the biosafety of AgNPs bioconjugates through haemolysis and cytotoxicity assays. As shown in Fig. 8a, there is a significant difference in haemolysis rates among various AgNPs. Compared to P-AgNPs, NP-AgNPs2 showed a lower haemolysis rate, which indicated that NP-AgNPs2 possessed a higher affinity for blood cells.

In order to test the cytotoxicity of the AgNPs samples, the viability of NIH-3T3 and HCT-116 cells treated with nisin, NP-AgNPs2, and P-AgNPs was determined. As shown in Fig. 8b, nisin demonstrated excellent cellular safety against NIH-3T3, with cell viability rates fell within the range of 92.2–95.2%, indicating that nisin was an AMP with high biocompatibility. Surprisingly, the cell viability rates for NIH-3T3 cells treated with NP-AgNPs2 were significantly higher than P-AgNPs. Notably, at the concentration of 0.4 mg/mL, the cell viability rates treated with NP-AgNPs2 and P-AgNPs reached 77.3 and 18.2%, respectively. These results indicated that nisin could significantly reduce the cytotoxicity of AgNPs, which was consistent with previous studies [34]. This could be explained that a nisin shell was formed on the surface of NP-AgNPs2, which prevented direct contact between AgNPs and NIH-3T3 cells. This reduction in non-specific interactions with mammalian cells inhibited the cytotoxicity of AgNPs [35]. Furthermore, the higher biocompatibility of nisin and less Ag^+^ release from NP-AgNPs2 might be another potential reasons for the reduced toxicity [12, 17].

As illustrated in Fig. 8c, nisin exhibited the ability to inhibit the proliferation of cancer cells HCT-116, and the cell viability decreased with the concentration increasement. Both NP-AgNPs2 and P-AgNPs demonstrated similar inhibitory effects on HCT-116 cells at higher concentrations. Compared with P-AgNPs, NP-AgNPs2 showed significant inhibitory effects on cancer cells at the concentration of 0.2 and 0.4 mg/mL. For example, at the concentration of 0.2 mg/mL, the viabilites of cancer cells treated with NP-AgNPs2 and P-AgNPs were 62.6% and 83.2%, respectively. This indicated that NP-AgNPs2 showed excellent antitumor properties even at low concentrations. This might be attributed to nisin’s specifically recognization and binding abilities to the surface of HCT-116 cells, thereby improving the targeting and antitumor property of AgNPs [36]. Additionally, the inherent antitumor activities of both nisin and AgNPs, along with their synergistic effects, may increase the lethality towards cancer cells [12, 17].

In short, NP-AgNPs reduced the toxicity of AgNPs against normal mammalian cells, but increased the inhibitory effect on cancer cells. These excellent properties will broaden the application of AgNPs in the medical field.

## Conclusions

This work employed a simple, green, and environmentally friendly method to synthesis bioconjugates NP-AgNPs, which exhibited a regular ellipsoidal shape and possessed a face-centred cubic crystal structure. FT-IR, XPS, and Zeta potential analysis indicated that nisin conjugated with AgNPs through electrostatic interactions, resulting in an increasement of surface potential. Antibacterial tests demonstrated that NP-AgNPs showed higher antibacterial performance than P-AgNPs. Antibacterial mechanism experiments revealed that NP-AgNPs deactivated bacteria by adhering to bacterial surfaces, disrupting cellular structures, and producing more ROS. Furthermore, NP-AgNPs exhibited excellent biosafety and antitumor efficacy. In short, NP-AgNPs exhibit excellent antibacterial properties, low cytotoxicity, and certain anti-cancer activity, and have great potential for application in the fields of food and medicine.

## Data Availability

The datasets generated during and/or analysed during the current study are available from the corresponding author on reasonable request.
